# Development of Multifunctional Liposomes Containing Magnetic/Plasmonic MnFe_2_O_4_/Au Core/Shell Nanoparticles

**DOI:** 10.3390/pharmaceutics11010010

**Published:** 2018-12-31

**Authors:** Ana Rita O. Rodrigues, Joana O. G. Matos, Armando M. Nova Dias, Bernardo G. Almeida, Ana Pires, André M. Pereira, João P. Araújo, Maria-João R. P. Queiroz, Elisabete M. S. Castanheira, Paulo J. G. Coutinho

**Affiliations:** 1 Centro de Física da Universidade do Minho (CFUM), Campus de Gualtar, 4710-057 Braga, Portugal; ritarodrigues@fisica.uminho.pt (A.R.O.R.); pg26303@alunos.uminho.pt (J.O.G.M.); pg36912@alunos.uminho.pt (A.M.N.D.); bernardo@fisica.uminho.pt (B.G.A.); ecoutinho@fisica.uminho.pt (E.M.S.C.); 2 IFIMUP/IN—Instituto de Nanociência e Nanotecnologia, Universidade do Porto, R. Campo Alegre, 4169-007 Porto, Portugal; ana.pires@fc.up.pt (A.P.); ampereira@fc.up.pt (A.M.P.); jearaujo@fc.up.pt (J.P.A.); 3 Centro de Química da Universidade do Minho (CQUM), Campus de Gualtar, 4710-057 Braga, Portugal; mjrpq@quimica.uminho.pt

**Keywords:** magnetic/plasmonic nanoparticles, multifunctional liposomes, manganese ferrite, gold shell, anti-tumor drugs, cancer therapy

## Abstract

Multifunctional liposomes containing manganese ferrite/gold core/shell nanoparticles were developed. These magnetic/plasmonic nanoparticles were covered by a lipid bilayer or entrapped in liposomes, which form solid or aqueous magnetoliposomes as nanocarriers for simultaneous chemotherapy and phototherapy. The core/shell nanoparticles were characterized by UV/Visible absorption, X-Ray Diffraction (XRD), Transmission Electron Microscopy (TEM), and Superconducting Quantum Interference Device (SQUID). The magnetoliposomes were characterized by Dynamic Light Scattering (DLS) and TEM. Fluorescence-based techniques (FRET, steady-state emission, and anisotropy) investigated the incorporation of a potential anti-tumor drug (a thienopyridine derivative) in these nanosystems. The core/shell nanoparticles exhibit sizes of 25 ± 2 nm (from TEM), a plasmonic absorption band (λ_max_ = 550 nm), and keep magnetic character. XRD measurements allowed for the estimation of 13.3 nm diameter for manganese ferrite core and 11.7 nm due to the gold shell. Aqueous magnetoliposomes, with hydrodynamic diameters of 152 ± 18 nm, interact with model membranes by fusion and are able to transport the anti-tumor compound in the lipid membrane, with a high encapsulation efficiency (*EE* *(%)* = 98.4 ± 0.8). Solid magnetoliposomes exhibit hydrodynamic diameters around 140 nm and also carry successfully the anticancer drug (with *EE (%)* = 91.2 ± 5.2), while also being promising as agents for phototherapy. The developed multifunctional liposomes can be promising as therapeutic agents for combined chemo/phototherapy.

## 1. Introduction

In recent years, a revolution in cancer therapy has taken place due to the development of multi-tasked nanostructures or materials for applications in oncology [1,2]. In chemotherapy, the ideal nano-encapsulation system should have biophysical properties that favor the passive accumulation in tumors upon intravenous administration, as well as controlled triggered release of the encapsulated active molecules. In this context, magnetic nano-encapsulation systems are promising since they can enable the magnetic drug targeting by static gradient magnetic fields and magnetic hyperthermia, which produce local heat as a trigger for drug release and a synergistic cytotoxic effect in cancer cells [3,4,5,6,7]. Additionally, systems based on superparamagnetic nanoparticles can generate high-resolution images by T2-weighted magnetic resonance imaging (MRI) for tumor diagnosis [7,8,9]. 

Noble metal (Ag, Au) nanoparticles strongly absorb light in the visible region due to coherent oscillations of the metal conduction band electrons in strong resonance with visible frequencies of light. This phenomenon is known as surface plasmon resonance (SPR) [10,11,12,13] and is highly dependent on nanoparticles size, shape, surface, and dielectric properties of the surrounding medium [14,15,16]. Light absorbed by nanoparticles is readily dissipated as heat. Due to their large absorption cross sections, plasmonic nanoparticles can generate a significant amount of heat and increase temperatures in their vicinities [17]. If a sufficient number of nanoparticles are present, the temperature fields overlap and create a substantial global temperature rise [18]. From the point of view of cancer therapeutics, noble metal nanoparticles become very useful as agents for plasmonic photothermal therapy (PTT) on account of their enhanced absorption cross sections, which are four to five orders of magnitude larger than those offered by conventional photo-absorbing dyes [16]. This strong absorption ensures effective laser therapy at relatively lower energies, which render the therapy method minimally invasive. Additionally, metal nanostructures have a higher photo-stability and do not suffer from photo-bleaching [16,19]. Recently, plasmonic nanoparticles have also been used as photoacoustic imaging (PAI) agents to increase tissue penetration, as well as sensitivity and spatial resolution [20].

In nanomedicine, systems with combined magnetic and plasmonic properties are of particular interest for theranostics since they combine simultaneously multiple imaging modalities for diagnosis with complementary synergistic strategies for therapy [21,22,23]. Gold nanoparticles have been largely used in biomedical applications for their low toxicity, great biocompatibility, easy conjugation with active biomolecules, and their remarkable optical properties, which enable their use as diagnostic and therapeutic agents [19,24]. However, recent works have shown that the conjugation of gold nanoparticles with magnetic ones may decrease the overall magnetization of the nanostructure [25]. Thus, coating magnetic nanoparticles with gold should be carefully considered in order to ensure proper magnetic capabilities for their application. Among all magnetic nanoparticles, those of manganese ferrite have recently received great attention for their high magnetic susceptibility, which suggests that they may be promising as hyperthermia and magnetic drug targeting agents [26,27].

In this study, magnetic/plasmonic nanoparticles possessing a manganese ferrite core and a gold shell were prepared. In order to develop applications in cancer therapy, the prepared nanoparticles were entrapped in liposomes (aqueous magnetoliposomes, AMLs) or covered with a lipid bilayer (solid magnetoliposomes, SMLs). These new nanosystems were tested in this scenario as nanocarriers for a potential anticancer drug, especially active against melanoma, breast adenocarcinoma, and non-small cell lung cancer [28]. In addition, the local heating capability of the developed systems was monitored through the fluorescence quenching of rhodamine B incorporated in the lipid layer when excited with a light source. Considering their potentialities, the new nanosystems developed in this study can be promising for future applications in cancer therapy.

## 2. Materials and Methods 

All the solutions were prepared using spectroscopic grade solvents and ultrapure water of Milli-Q grade (MilliporeSigma, St. Louis, MO, USA). 

### 2.1. Preparation of Manganese Ferrite/Gold Core/Shell Nanoparticles

Manganese ferrite nanoparticles (NPs) were synthesized in 5 mL aqueous solution, by the co-precipitation method, as previously described [26]. First, an aqueous solution containing 612 μL of 50% NaOH solution was heated to 90 °C. Then, a mixture containing 500 μL of 0.5 M MnSO_4_·H_2_O solution and 500 μL of 1 M FeCl_3_·6H_2_O solution was added, drop by drop, to the previously warmed basic solution under magnetic stirring. After two hours at 90 °C, manganese ferrite nanoparticles were formed. For purification, the obtained sample was washed several times with ethanol, by centrifugation (14,000 *g*) and magnetic decantation.

For growth of the gold shell, a method adapted from a previously described procedure was used [19]. In addition, 5 mL of an aqueous dispersion of the synthesized MnFe_2_O_4_ nanoparticles (with concentration of 4 mg/mL) were added to 25 mL of glycerol and heated up to 200 °C, under vigorous stirring. Then, 2 mL of 0.02 M solution of gold(III) chloride hydrate (HAuCl_4_), from Sigma-Aldrich (St. Louis, MO, USA), were added dropwise. After 15 minutes under continuous stirring at 200 °C, the gold shell was formed around the MnFe_2_O_4_ core NPs. To remove glycerol residues, the synthesized NPs were washed by centrifugation (14,000 *g*) with ethanol.

### 2.2. Preparation of Magnetoliposomes

For magnetoliposomes preparation, the lipids l*-*α*-*phosphatidylcholine from egg yolk (Egg-PC), and 1,2-dioleoyl-*sn*-glycero-3-phospho-*rac*-(1-glycerol) sodium salt (DOPG), from Sigma-Aldrich (St. Louis, MO, USA), were used in a final concentration of 1 mM. The ethanol injection method was employed to obtain aqueous magnetoliposomes (AMLs) [29]. Accordingly, a 20 mM lipid solution in ethanol was injected, under vigorous vortexing, to an aqueous dispersion of manganese ferrite/gold nanoparticles (with 4 mg/mL concentration). After encapsulation, the ferrofluid was washed with water and purified by magnetic decantation to remove all the non-encapsulated NPs.

For the preparation of solid magnetoliposomes (SMLs), a method previously described was used [30]. First, 10 μL of a solution of the synthesized MnFe_2_O_4_/Au core/shell nanoparticles (0.02 mg/mL) were ultra-sonicated for one minute at 189 W, and 3 mL of chloroform were added to the solution. Then, immediately after vigorous agitation, 150 μL of a 20 mM methanolic solution of the lipid DOPG (1,2-dioleoyl-*sn*-glycero-3-phospho*-rac*-(1-glycerol) sodium salt) were injected under vortexing to form the first lipid layer of the SMLs. To remove the lipid that was not attached to the nanoparticles surface, the particles were washed twice by magnetic decantation with ultrapure water. The lipid bilayer was completed by a new injection of 150 μL of 20 mM lipid methanolic solution, under vortexing, in 3 mL of aqueous dispersion of the particles with the first lipid layer. The SMLs obtained were then washed and purified with ultrapure water by magnetic decantation.

The anti-tumor compound methyl 3-amino-6-(benzo[*d*]thiazol-2-ylamino)thieno[3,2-*b*]pyridine-2-carboxylate was incorporated into aqueous magnetoliposomes by the co-injection method (simultaneous injection of compound and lipid) in a final compound concentration of 2 μM. In solid magnetoliposomes, the compound was incorporated by injection of an ethanolic solution (0.2 mM) immediately before the formation of the second lipid layer.

### 2.3. Preparation of Giant Unilamellar Vesicles (GUVs)

GUVs of soybean lecithin (l-α-phosphatidylcholine from soybean), from Sigma-Aldrich (St. Louis, MO, USA), were obtained by the thin film hydration method [31,32]. For that, a lipid film of 100 μL of soybean lecithin solution (1 mM) was obtained by solvent evaporation under an argon stream, and 40 μL of water were added, followed by incubation at 45 °C for 30 min. Then, 3 mL of glucose aqueous solution (0.1 M) were added and the resulting solution was again incubated at 37 °C for 2 h. After incubation, the GUVs suspension was centrifuged at 14,000 *g* for 30 minutes at 20 °C, to remove multi-lamellar vesicles and lipid aggregates.

### 2.4. Spectroscopic Measurements

#### 2.4.1. General Methods 

Absorption spectra were performed in a Shimadzu UV-3600 Plus UV-vis-NIR (Shimadzu Corporation, Kyoto, Japan) spectrophotometer. Fluorescence measurements were recorded using a Horiba Fluorolog 3 spectrofluorimeter (HORIBA Jobin Yvon IBH Ltd., Glasgow, UK), equipped with double mono-chromators in both excitation and emission, Glan-Thompson polarizers, and a temperature controlled cuvette holder. Fluorescence spectra were corrected for the instrumental response of the system. 

#### 2.4.2. FRET Measurements

Förster Resonance Energy Transfer (FRET) assays were employed to confirm the formation of the lipid bilayer in the solid magnetoliposomes (SMLs). For that purpose, the nitrobenzoxazole labeled lipid NBD-C_6_-HPC (1-palmitoyl-2-{6-[(7-nitro-2-1,3-benzoxadiazol-4-yl)amino]hexanoyl} -*sn*-glycero-3-phosphocholine) (from Avanti Polar Lipids, Alabaster, AL, USA) was included in the first lipid layer, while the rhodamine B labeled lipid Rhodamine B-DHPE (1,2-dipalmitoyl-*sn*-glycero-3-phospho-ethanolamine-*N*-lissamine rhodamine B sulfonyl (ammonium salt)) (from Avanti Polar Lipids, Alabaster, AL, USA) was included in the second lipid layer.

FRET efficiency, Φ_RET_, defined as the proportion of donor molecules that have transferred their excess energy to the acceptor molecules, was calculated through donor emission quenching, by taking the ratio of the donor integrated fluorescence intensities in the presence of acceptor (F_DA_) and in the absence of acceptor (F_D_) (Equation (1)) [33].

(1) $$\Phi_{\mathrm{RET}}=\;1\;-\;\frac{F_{\mathrm{DA}}}{F_{D}}$$

The distance between the donor and acceptor molecules was determined through the FRET efficiency (Equation (2)).

(2) r= R0[1−ΦRETΦRET]16

where *R*_0_ is the Förster radius (critical distance), that can be obtained by the spectral overlap, *J*(*λ*), between the donor emission and the acceptor absorption, according to Equations (3) and (4) (with *R*_0_ in Å, *λ* in nm, *ε_A_*(*λ*) in M^−1^ cm^−1^) [33].

(3) R0 =0.2108[k2ΦD0n−4J(λ)]16

(4) $$J\left(\;\lambda \right)=\;\int_{0}^{\;\infty }I_{D}(\lambda )\;\varepsilon_{A}\left(\lambda \right)\;\lambda^{4}d\lambda$$

where k2=23 is the orientational factor assuming random orientation of the dyes, *n* is the refraction index of the medium, *I_D_*(*λ*) is the fluorescence spectrum of the donor normalized so that $\int_{0}^{\;\infty }I_{D}\left(\lambda \right)d\lambda =1$, and *ε_A_*(*λ*) is the molar absorption coefficient of the acceptor. $\Phi_{D}^{0}$, the fluorescence quantum yield of the donor in the absence of energy transfer, was determined by the standard method (Equation (5)) [34,35].

(5) $$\Phi_{D}^{0}=\frac{A_{r}F_{D}n_{D}^{2}}{A_{D}F_{r}n_{r}^{2}}\Phi_{r}$$

where *A* is the absorbance at the excitation wavelength, *F* is the integrated emission area, and *n* is the refraction index of the solvents used. Subscripts refer to the reference (r) or donor (D). The absorbance at the excitation wavelength was always lower than 0.1 to avoid the inner filter effects. The NBD-C_6_-HPC molecule intercalated in lipid membranes was used as a reference, Φ_r_ = 0.32 at 25 °C, as reported by Invitrogen [36].

The hydrophobic dye Nile Red (energy acceptor) was also incorporated in magnetoliposomes labelled with NBD-C_6_-HPC (NBD as energy donor) for monitoring the interaction of magnetoliposomes with GUVs by FRET.

#### 2.4.3. Fluorescence Anisotropy Measurements

The steady-state fluorescence anisotropy, *r*, is calculated by the equation below.

(6) $$r=\frac{I_{\mathrm{VV}}-GI_{\mathrm{VH}}}{I_{\mathrm{VV}}+2GI_{\mathrm{VH}}}$$

where *I*_VV_ and *I*_VH_ are the intensities of the emission spectra obtained with vertical and horizontal polarization, respectively (for vertically polarized excitation light), and $G=I_{\mathrm{HV}}/I_{\mathrm{HH}}$ is the instrument correction factor, where *I*_HV_ and *I*_HH_ are the emission intensities obtained with vertical and horizontal polarization (for horizontally polarized excitation light).

#### 2.4.4. Drug Encapsulation Efficiency

The encapsulation efficiency, *EE* (%), of the potential anti-tumor drug in magnetoliposomes, was determined through fluorescence emission measurements. Therefore, drug loaded magnetoliposomes were subjected to centrifugation at 11,000 rpm for 60 min using Amicon^®^ Ultra centrifugal filter units 100 kDa (Merck Millipore, Darmstadt, Germany). Then, the filtrate (containing the non-encapsulated drug) was pipetted out, the water was evaporated, and the same amount of ethanol was added. After vigorous agitation, its fluorescence was measured, which allowed it to determine the drug concentration using a calibration curve (fluorescence intensity *vs.* concentration) previously obtained in the same solvent. Three independent measurements were performed for each system and standard deviations (SD) were calculated. The encapsulation efficiency was determined using Equation (7).

(7) $$EE\left(\%\right)=\;\frac{\left(total\;amount-amount\;of\;non\;encapsulated\;compound\right)}{total\;amount}\times 100$$

### 2.5. Structural Characterization

#### 2.5.1. Transmission Electron Microscopy (TEM)

TEM images of nanoparticles and solid magnetoliposomes were acquired using a Transmission Electron Microscope Leica LEO 906E (Leica Microsystems, Wetzlar, Germany) operating at 120 kV, at UME (Electron Microscopy Unit), University of Trás-os-Montes and Alto Douro (Vila Real, Portugal). For SMLs, a negative staining was employed, using a 2% aqueous solution of ammonium molybdate tetrahydrate. In addition, 20 μL of the sample and 20 μL of the staining solution were mixed and a drop of the mixture was placed onto a Formvar grid (Agar Scientific Ltd., Essex, UK), held by tweezers. After 20 s, almost all the solution was removed with filter paper and left to dry. TEM images were processed using *ImageJ* software (National Institutes of Health (NIH), Bethesda, MD, USA) with the addition of a value to all pixels so that a white background resulted, which was followed by inversion and enhanced local contrast. Subsequently, the ParticleSizer plugin [37] was used and was followed by particle analysis. The area of each particle allowed an estimation of the particle diameter. The resulting histogram was fitted to a bimodal Gaussian distribution.

#### 2.5.2. X-Ray Diffraction (XRD) 

X-Ray Diffraction (XRD) analyses were performed using a conventional Philips PW 1710 (Royal Philips, Amsterdam, The Netherlands) diffractometer, operating with CuK_α_ radiation, in a Bragg-Brentano configuration.

#### 2.5.3. Dynamic Light Scattering (DLS)

The mean diameter and size distribution (polydispersity index) of aqueous and solid magnetoliposomes (1 mM lipid concentration) were measured using Dynamic Light Scattering (DLS) equipment NANO ZS Malvern Zetasizer (Malvern Panalytical Ltd., Malvern, UK) at 25 °C, using an He-Ne laser of λ = 632.8 nm and a detector angle of 173°. The measurements were also carried out for the magnetoliposomes in a solution of human serum albumin (35 mg/mL) in PBS buffer (pH = 7.4). Five independent measurements were performed for each sample.

### 2.6. Magnetic Measurements

Magnetic measurements of the dry core/shell nanoparticles were performed at room temperature in a Superconducting Quantum Interference Device (SQUID) magnetometer Quantum Design MPMS5XL (Quantum Design Inc., San Diego, CA, USA) using applied magnetic fields up to 5.5 T.

### 2.7. Measurement of the Photothermal Effect

Solid magnetoliposomes incorporating the labelled lipid Rhodamine B-DHPE were irradiated and Rhodamine B emission was monitored by a function of time, using the detection system of a SPEX Fluorolog 2 spectrofluorimeter (HORIBA Jobin Yvon IBH Ltd., Glasgow, UK). The irradiation setup consisted in a Xenon arc lamp (200 W) and an optical fiber, using a Thorlabs FEL0600 (Thorlabs Inc., Newton, NJ, USA) long pass filter with cut-on wavelength at 600 nm, to ensure the excitation of only the gold nanoparticles (not exciting Rhodamine B dye).

## 3. Results and Discussion

### 3.1. Nanoparticles Characterization

#### 3.1.1. Absorption Spectra

**Figure 1:**
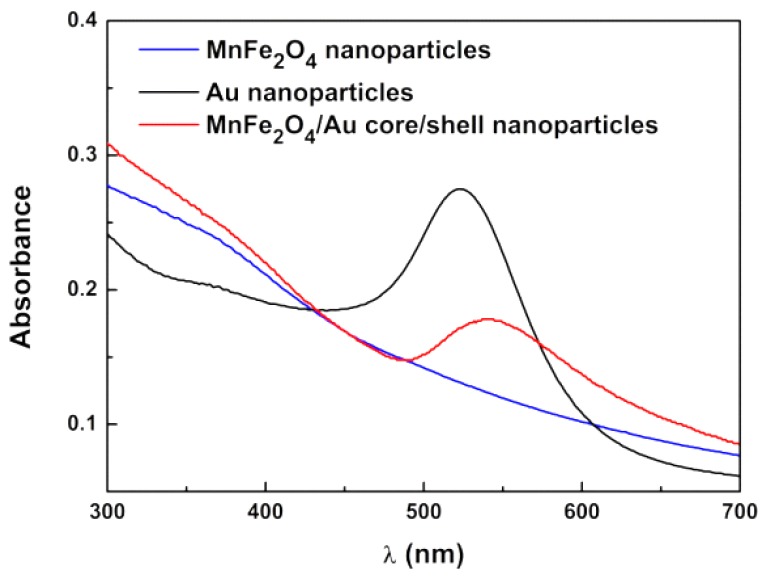
UV-Visible absorption spectra of aqueous dispersions of manganese ferrite nanoparticles, gold nanoparticles, and MnFe_2_O_4_/Au core/shell nanoparticles.

Figure 1 displays the UV-Visible absorption spectrum of the synthesized manganese ferrite/gold core/shell nanoparticles. The absorption spectra of net gold nanoparticles and net manganese ferrite nanoparticles are also shown for comparison. 

The spectrum of manganese ferrite NPs is typical of an indirect semiconductor, as reported earlier [26], while the spectrum of gold nanoparticles obtained by the standard Turkevish method [38] reveals a characteristic local surface plasmon resonance (LSPR) band, with a maximum around 530 nm. In comparison, the absorption spectrum of manganese ferrite/gold core/shell NPs exhibits a broader and red shifted plasmon band (maximum at 550 nm). The absorption spectrum of gold nanoshells depends on their thickness, as well as on the refraction index of both core and surrounding media [39]. Theoretical studies have shown that, for an air filled core of 10 nm size and a gold shell of 5 nm in water medium, the resonance peak is expected to occur at 538 nm, while, for a 10-nm shell, it should appear at 552 nm [40]. The increase of the core refraction index results in a red shift of the plasmon resonance peak [39]. For a 3-nm gold shell thickness on a 10-nm magnetite core, a resonance peak at 560 nm was found [41].

#### 3.1.2. X-Ray Diffraction (XRD) Measurements

**Figure 2:**
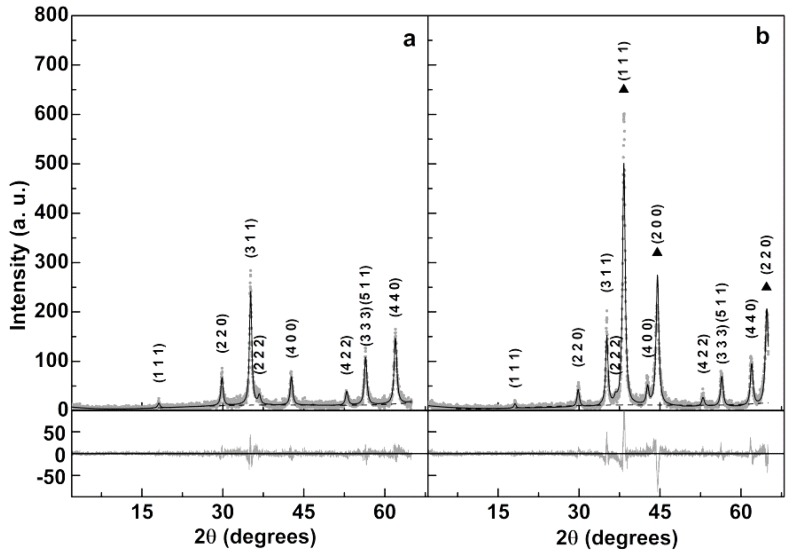
XRD diffractogram of manganese ferrite (**a**) and manganese ferrite/gold core/shell nanoparticles (**b**). Gold diffraction peaks are marked by a filled triangle.

XRD analysis confirmed the synthesis of a pure crystalline phase of manganese ferrite/gold nanoparticles, since all their characteristic peaks (CIF 2300618 for manganese ferrite and CIF 9013035 for gold), marked by their indices, were observed (Figure 2b). The percentage amounts obtained for MnFe_2_O_4_ and Au were 59.1% and 40.9%, respectively. Mean sizes of 13.3 nm for manganese ferrite and 11.7 nm for gold, were estimated through a Rietveld analysis using Fullprof software [42]. Table 1 summarizes the main results of the Rietveld analysis. For the net manganese ferrite powdered sample, it resulted in a poor R_F_ factor of 9.0. It was possible to improve it by optimizing the overall isothermal factor, B_over_, but an unreasonable value of −2.79 was obtained. A similar improvement was possible by accounting for the effect of sample microstructure (Figure 2a), according to Equation (8) [43], which gives the micro-absorption correction term, *P*.

(8) $$P=P_{0}+C\frac{\tau }{\sin\theta }\left(1-\frac{\tau }{\sin\theta }\right)$$

where *P*_0_ is the bulk contribution to the micro-absorption effect and *τ* is the normalized surface roughness parameter [43].

Considering $\mu \bar{l}=\;$0.01 (*μ* is the linear absorption coefficient and $\bar{l}$ is the mean chord length of the powder particle) and the values in Table 1 for Equation (8) parameters, a degree of inversion of *i* = 0.60 is obtained for manganese ferrite. This value is close to the one reported by Chen et al. (*i* = 0.67) [44], using a similar preparation method for MnFe_2_O_4_. The Rietveld analysis of gold phase was not optimal, as the intensity of peak (111) is lower and that of peak (200) is higher than the experimental ones. In addition, the analysis of MnFe_2_O_4_ phase decreased its quality, as the R_F_ factor increased from 3.18 to 4.70 and the peak (311) got much lower than the experimental one. This could be due to the expected shell morphology of gold in the prepared MnFe_2_O_4_/Au nanocomposites in which a layer of gold grows on the MnFe_2_O_4_ surface. This morphology, through lattice mismatch induced stress, is predictable to change the intensity of the diffraction peaks [45]. An atomistic modelling of the core/shell nanoparticle is anticipated to yield a better description of the XRD diffractogram [46] and this will be addressed in a future study. The obtained weight fraction of gold was 40.9%, but this is calculated using proportionality factors between diffraction intensity and mass, ATZs [42], that do not take into account the dependence of diffraction intensity on particle size. Since the lattice constant of Au is less than half that of MnFe_2_O_4_, the reduction of diffraction intensity with particle size is much more pronounced for Au than for MnFe_2_O_4_. This means that the value given by Fullprof software is expected to be much lower than the real one. Considering the obtained size of manganese ferrite of 13.3 nm and using the phase densities that resulted from the XRD analysis (5.04 g cm^−3^ for MnFe_2_O_4_ and 19.4 g cm^−3^ for Au), the mass percentage of gold would be 98.7% if the obtained size of gold phase (11.7 nm) corresponds to the shell thickness. This percentage would change to 95.6% if the obtained size value of 11.7 nm corresponds to the double of the shell thickness. This would be true if the effect of the two gold layers in given X-ray crosses contributes equally to the broadening of the diffraction peak. Thus, from XRD data analysis, the thickness of the gold shell is expected to be 5.85 nm. This value is compatible with the observed position of the surface plasmon resonance peak, as discussed in the previous section. Additionally, in magnetite/gold core/shell nanoparticles, reported in Reference [41], the diffraction peak widths were identical for the 10 nm magnetite core and for the 2 nm gold shell (where its dimensions were obtained from HR-TEM measurements). Therefore, the actual value of the gold shell thickness in MnFe_2_O_4_/Au NPs could be even lower.

A nanostructure consisting of a 5.85 nm gold shell surrounding a cluster of MnFe_2_O_4_ nanoparticles cannot be ruled out. In that case, the calculated mass percentage of gold changes to 91% for a compact over-coating layer of 12 spheres. Nevertheless, in the case of gold shell growth, the magnetic nanoparticles are dispersed in glycerol at a high temperature, before the addition of HAuCl_4_ solution. This means the magnetic nanoparticles can effectively be dispersed with their surface well stabilized and passivated by the abundant OH groups of glycerol. Furthermore, the formation of the gold shell occurs through oxidation of glycerol. This process originates in other molecules, such as glyceric acid or tartronic acid, which can act as gold surface stabilizers. This is the case in terms of the citric acid in the Turkevish gold nanoparticles synthesis procedure [38]. Therefore, the prepared MnFe_2_O_4_/Au core/shell nanoparticles are expected to be surface passivated by hydroxyacids and, as such, well dispersible in aqueous media.

#### 3.1.3. Transmission Electron Microscopy (TEM)

**Figure 3:**
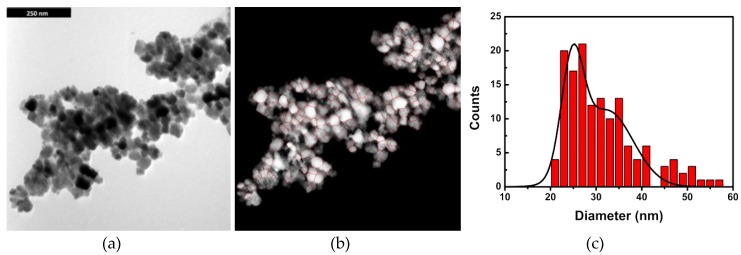
(**a**) TEM image of the synthesized MnFe_2_O_4_/Au core/shell nanoparticles. (**b**) TEM image processed by *ImageJ* (same scale of image **a**). (**c**) Particles size histogram and fitting to a bimodal Gaussian distribution (total number of 141 particles).

TEM images (Figure 3a) of the MnFe_2_O_4_/Au prepared nanoparticles and the corresponding image after processing by *ImageJ* (Figure 3b) revealed a generally spherical shape with the presence of some aggregation. These aggregates probably arise from the sample preparation on TEM grids (slow evaporation of a drop of an aqueous dispersion of nanoparticles). The size histogram that results from the area of the highlighted particles was fitted to a bimodal Gaussian distribution in order to better separate the presence of aggregates from the individual nanoparticles (Figure 3c). A bimodal size distribution of 25 ± 2 nm and 32 ± 6 nm was obtained. The former population is in accordance with the size estimated from XRD measurements, which is 25 nm when the gold shell thickness is 5.85 nm.

Below is a critical diameter of 42.9 nm. MnFe_2_O_4_ nanoparticles possess a superparamagnetic behavior [47,48], losing at least 90% of the magnetization when an applied magnetic field is removed, which is important for biomedical applications. The size of the nanoparticles obtained in this study is within this limit, and, therefore, these NPs are suitable for applications in biomedicine.

### 3.2. Magnetic Properties 

**Figure 4:**
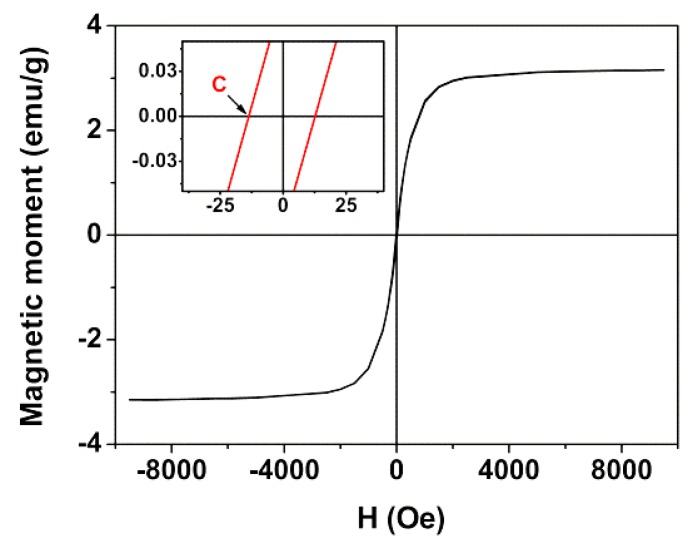
Magnetization hysteresis loop of MnFe_2_O_4_/Au core/shell nanoparticles measured at room temperature. Inset: Enlargement of the loop in the low field region.

The magnetic properties of MnFe_2_O_4_/Au core/shell nanoparticles (Figure 4) were characterized by measuring their magnetic hysteresis loop, which shows the relationship between the induced magnetic moment and the applied magnetic field (*H*). The core/shell nanoparticles present a superparamagnetic behavior since the ratio between remnant magnetization (*M*_r_) and saturation magnetization *(M_s_*) is below 0.1 [49] (Table 2).

The low saturation magnetization values are due to the presence of a diamagnetic gold layer. The gold shell thickness of MnFe_2_O_4_/Au core/shell nanoparticles was estimated using the magnetic hysteresis cycle. The particles were considered to have a well ordered MnFe_2_O_4_ core covered by a non-magnetic gold shell (with a thickness *δ*), acting as a “dead layer”. Thus, the obtained saturation magnetization, *M*_s_, is proportional to the core volume that possesses a spontaneous magnetization, which is related to the thickness of the dead layer and to the particle diameter, through Equation (9) [49].

(9) $$M_{s}=\;M_{s0\;}\left(1-\frac{6\delta }{D}\right)$$

where *D* is the particle diameter and *M*_s0_ is the saturation magnetization of MnFe_2_O_4_.

Chen et al. [44] observed that the saturation magnetization of manganese ferrite depends on particle size, which is estimated to be a value of 58 emu/g for MnFe_2_O_4_ nanoparticles of 13.3 nm (also prepared by coprecipitation). Using this value, a gold layer thickness of *δ* = 4.0 nm is obtained for particles with a diameter of 25 nm (from TEM) and *M*_s_ = 3.15 emu/g. This is roughly in accordance with XRD results.

### 3.3. Magnetoliposomes as Drug Nanocarriers

**Figure 5:**
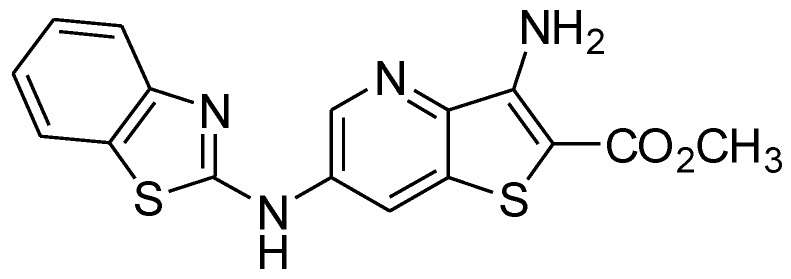
Structure of the potential anti-tumor thienopyridine derivative.

The obtained magnetic/plasmonic nanoparticles were either entrapped in liposomes (aqueous magnetoliposomes, AMLs) or covered by a lipid bilayer that forms the so-called solid magnetoliposomes (SMLs). The potential of both types of magnetoliposomes as drug carriers was investigated. A potential anti-tumor compound, which is a fluorescent thienopyridine derivative (Figure 5), was incorporated into AMLs and SMLs and its fluorescence emission was studied. This compound can be promising as an anticancer agent in oncological therapy, as it exhibited very low growth inhibitory concentrations (GI_50_), between 3.5 μM (for A375-C5 melanoma cell line) and 6.4 μM (for non-small cell lung cancer, NCI-H460 cell line), when tested in vitro in several human tumor cell lines [28]. Moreover, the same compound has shown a very low affinity for the multi-drug resistance protein (MDR1), which is a protein that promotes drug resistance in cells [50]. This compound exhibits fluorescence in several polar and non-polar media, but not in aqueous solution [50]. Therefore, fluorescence-based methodologies (fluorescence emission, FRET, and fluorescence anisotropy) are advantageous techniques to monitor behavior and location of this compound in magnetoliposomes.

#### 3.3.1. Aqueous Magnetoliposomes

**Figure 6:**
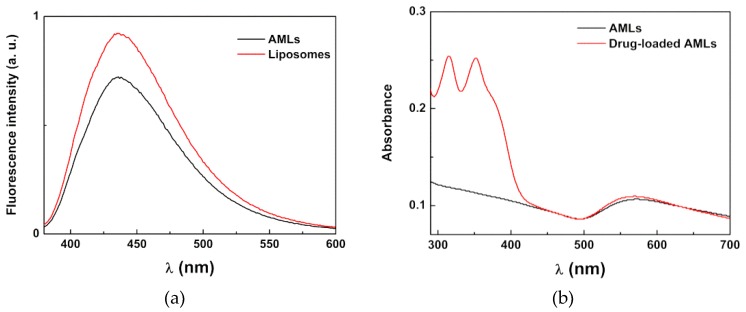
(**a**) Fluorescence spectra (λ_exc_ = 360 nm) of the anti-tumor compound (2 × 10^−6^ M) in liposomes and AMLs of Egg-PC containing MnFe_2_O_4_/Au core/shell nanoparticles; (**b**) Absorption spectra of AMLs containing the core/shell nanoparticles with and without the anti-tumor drug.

The emission of the thienopyridine derivative in AMLs and liposomes (the latter without nanoparticles and with the same concentration of compound) is shown in Figure 6a. Since this potential drug is not fluorescent in aqueous media [50], the emission observed is indicative of the encapsulation of the compound in magnetoliposomes. A quenching of the compound fluorescence is observed in AMLs relative to the liposomes, which confirms its incorporation in the magnetic nanocarriers since this effect is attributed to the presence of the nanoparticles that absorb in a wide wavelength range [26]. Figure 6b displays the absorption spectra of AMLs containing the core/shell nanoparticles, with and without the anti-tumor drug, which confirms the successful loading of the drug into the magnetoliposomes and shows the overlap of the absorption spectra of nanoparticles with the compound fluorescence emission. This indicates that the nanoparticles can quench, by energy transfer, the compound fluorescence, as already observed [26]. Additionally, the gold shell can also introduce a quenching effect through electron transfer processes.

Moreover, the fluorescence anisotropy measurements (Table 3) confirmed that the anti-tumor compound is located mainly in the lipid bilayer. The anisotropy values were analogous to those previously determined in liposomes of the same lipids [50]. The behavior observed is similar to the one previously reported for magnetoliposomes containing manganese ferrite nanoparticles (without the gold shell) [26], which indicates that gold does not influence compound location in magnetoliposomes.

The size of DOPG AMLs containing the magnetic/plasmonic nanoparticles were determined by Dynamic Light Scattering (DLS), which exhibit hydrodynamic diameters of 152 ± 18 nm and with a low polydispersity index (PDI = 0.19 ± 0.04). This size is larger than the one reported for Egg-PC aqueous magnetoliposomes containing manganese ferrite nanoparticles [26], but very similar to the one of DOPG solid magnetoliposomes based on MnFe_2_O_4_ [26]. In the presence of human serum albumin (HSA), 35 mg/mL (typical concentration in serum) in PBS buffer, the size of AMLs slightly increases and possesses hydrodynamic diameters of 157 ± 29 nm with a slightly higher polydispersity (PDI = 0.23 ± 0.09). For enhanced permeability and a retention (EPR) effect of loaded drugs, the diameter of (magneto)liposomes must be small. A successful extravasation into tumors has been shown to occur for nanocarriers with sizes below 200 nm [51]. Moreover, an encapsulation efficiency of *EE (%)* = 98.4 ± 0.8 was obtained for the anti-tumor thienopyridine derivative in these aqueous magnetoliposomes, which anticipate that these systems contain MnFe_2_O_4_/Au NPs as very promising nanocarriers for anti-cancer drugs. 

**Figure 7:**
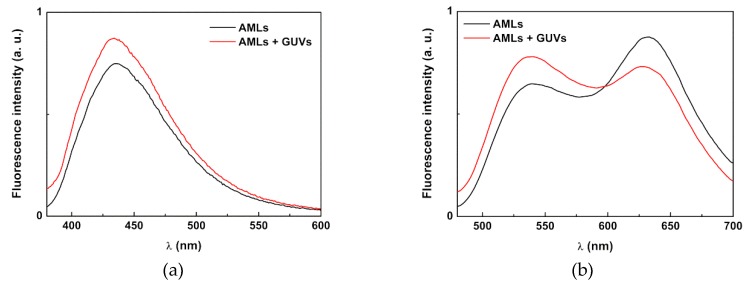
(**a**) Fluorescence spectra (λ_exc_ = 360 nm) of the thienopyridine derivative (2 × 10^−6^ M) in AMLs of Egg-PC containing MnFe_2_O_4_/Au nanoparticles, before and after interaction with GUVs. (**b**) Fluorescence spectra (λ_exc_ = 400 nm) of AMLs containing both NBD-C_6_-HPC (2 × 10^−6^ M) and Nile Red (2 × 10^−6^ M), before and after interaction with GUVs.

The interaction of AMLs containing MnFe_2_O_4_/Au nanoparticles with Giant Unilamellar Vesicles (GUVs), used as models of biological membranes, was also investigated. The aim of this study was to evaluate the ability of magnetoliposomes to release drugs by fusing with cell membranes, considering future applications in cancer therapy/theranostics. Taking into account the fluorescence quenching caused by the presence of the core/shell nanoparticles, the emission of the anti-tumor compound incorporated in aqueous magnetoliposomes was measured before and after interaction with GUVs (Figure 7a). After interaction with GUVs, the observed unquenching effect indicates the occurrence of membrane fusion between AMLs and the model membranes, with an increase in the distance between the drug and the nanoparticles (decreasing the interaction that leads to emission quenching). 

To further confirm that this unquenching is, in fact, due to fusion between AMLs and GUVs, the interaction between these two systems was also monitored by FRET (Förster Resonance Energy Transfer). For that purpose, AMLs containing both the labeled lipid NBD-C_6_-HPC and the lipid probe Nile Red [52,53,54] were prepared. In this case, the NBD moiety acted as the energy donor and the dye Nile Red acted as the energy acceptor [55]. Exciting only the donor NBD, a strong band due to Nile Red emission was observed (with maximum around 630 nm), which resulted from energy transfer to Nile Red (Figure 7b). After interaction with GUVs, the donor fluorescence (λ_max_ = 535 nm) increased and the acceptor emission band decreased, which showed the diminution of FRET process efficiency and, consequently, proved the membrane fusion between AMLs and GUVs.

#### 3.2.2. Solid Magnetoliposomes

The preparation of solid magnetoliposomes, where a cluster of nanoparticles is successively covered by two lipid layers, was performed using a methodology previously developed [30]. This method has proven to be successful for solid magnetoliposomes containing magnetic nanoparticles of manganese ferrite [26], nickel ferrite [30], and magnetite [56]. To confirm that the same procedure can be applied to nanoparticles with a gold shell, the formation of the lipid bilayer around MnFe_2_O_4_/Au nanoparticles was confirmed by FRET.

**Figure 8:**
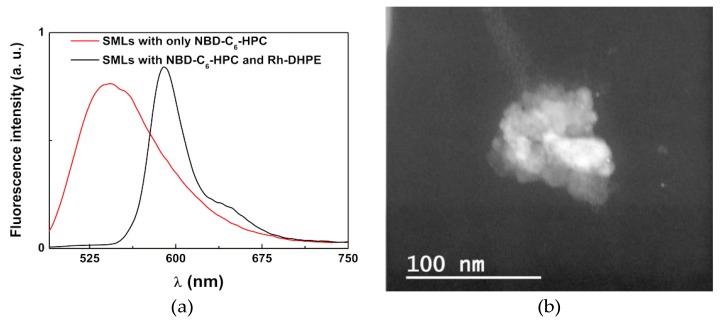
(**a**) Fluorescence spectra (λ_exc_ = 470 nm, no rhodamine excitation) of SMLs of DOPG containing MnFe_2_O_4_/Au core/shell nanoparticles labeled with only NBD-C_6_-HPC and labeled with both NBD-C_6_-HPC and rhodamine B-DHPE; (**b**) TEM image (dark field mode) of SMLs containing core/shell nanoparticles (obtained with a negative staining).

The labeled lipid NBD-C_6_-HPC (NBD as energy donor) was included in the first lipid layer of the SMLs and the lipid Rhodamine B-DHPE (rhodamine as the acceptor) was included in the second lipid layer. The emission of SMLs containing only the NBD-labelled lipid and SMLs containing both donor and acceptor labeled lipids were measured (Figure 8a), exciting only the donor NBD.

The fluorescence spectrum of SMLs with only the donor shows, as expected, a characteristic NBD emission band (λ_max_ = 535 nm). On the other hand, the fluorescence spectrum of the SMLs containing both donor and acceptor rates reveals a decrease in the NDB emission opposing the strong rise in the rhodamine B emission band, which shows the energy transfer of the excited NBD moiety to rhodamine B. A distance between donor and acceptor of r_DA_ = 3.1 nm was determined, from the calculated FRET efficiency of 0.65 (Equations (2)–(5)). Considering that donor and acceptor are each in one of the lipid layers, the distance between them proves the bilayer formation in SMLs, considering the typical cell membrane thickness (7–9 nm) [57]. TEM images of solid magnetoliposomes (Figure 8b) containing the core/shell nanoparticles point to nanosystems with diameters around 100 nm. DLS measurements revealed that these solid liposomes exhibit hydrodynamic diameters of 138 ± 19 nm and a low value for the polydispersity index (PDI = 0.20 ± 0.07). In the presence of HSA (PBS buffer, pH = 7.4), the size determined by DLS rises to 160 ± 32 nm (PDI = 0.24 ± 0.08), but is still below 200 nm.

**Figure 9:**
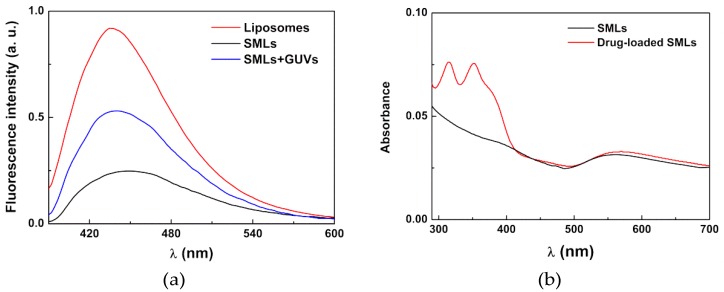
(**a**) Fluorescence spectra (λ_exc_ = 360 nm) of the thienopyridine derivative (2 × 10^−6^ M), in SMLs of DOPG containing MnFe_2_O_4_/Au core/shell nanoparticles, before and after interaction with GUVs. (**b**) Absorption spectra of SMLs containing the core/shell nanoparticles, with and without the anti-tumor drug.

The incorporation of the anti-tumor thienopyridine derivative in SMLs and the fusion with model membranes (GUVs) were confirmed in a similar way to the AMLs. As such, the emission of compounds loaded in SMLs was measured, as well as the emission after interaction of the SMLs with GUVs (Figure 9a). It was observed that a quenching effect of the compound fluorescence emission by the presence of magnetic/plasmonic nanoparticles (relative to the observed in liposomes) indicates incorporation of the thienopyridine derivative in the SMLs membrane. This quenching is more pronounced than for AMLs due to the lower distance between the NPs cluster and the drug. Figure 9b shows the absorption spectra of drug loaded and unloaded SMLs. Like in the case of AMLs, SMLs can also absorb (through the core/shell nanoparticles) in the compound emission region.

The fluorescence anisotropy value of the anti-tumor drug in these SMLs is also similar to the one in liposomes and AMLs of the same lipid (Table 3). This indicates that the main location of the compound is in the lipid membrane, as reported for liposomes [50]. The drug encapsulation efficiency in SMLs is slightly lower than the one for AMLs, *EE (%)* = 91.2 ± 5.2, but still quite high.

The unquenching effect detected upon interaction with GUVs proves membrane fusion of SMLs with the model membranes. Again, these results are similar to the ones in magnetoliposomes containing net MnFe_2_O_4_ nanoparticles [26] and, hence, the SMLs containing magnetic/plasmonic nanoparticles are promising nanocarriers for this anti-tumor drug.

### 3.4. Magnetoliposomes as Agents for Phototherapy 

**Figure 10:**
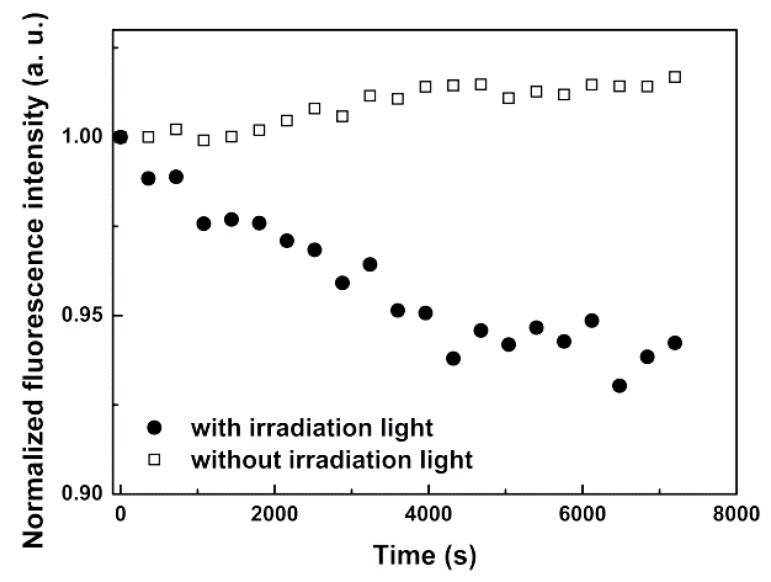
Fluorescence intensity (normalized to initial intensity) of irradiated SMLs with MnFe_2_O_4_/Au core/shell nanoparticles labelled with Rhodamine B-DHPE, as a function of time. For comparison, the behavior without irradiation light is also shown.

For the study of photo thermal ability, solid magnetoliposomes containing MnFe_2_O_4_/Au core/shell nanoparticles and including the labeled lipid Rhodamine B-DHPE were synthesized and Rhodamine B emission was measured under irradiation (λ > 600 nm) along time (Figure 10). The local heating produced by gold propagates by heat diffusion, resulting in quenching of the rhodamine emission, due to the increase of efficiency of the non-radiative decay pathways. This effect is observed in Figure 10, where a monotonic decrease in Rhodamine fluorescence intensity is detected along the irradiation time, which leads to a local temperature increase. The solution temperature raised only 2 °C during irradiation time, but this increase also occurred in the absence of irradiation. This indicates that the excitation light from the spectrofluorometer, which is always incident in the sample during measurement, has higher intensity than the irradiation light (at λ > 600 nm), so that the effect of the latter is negligible. When only the excitation light hits the sample, the rhodamine emission intensity is approximately constant (Figure 10). On the contrary, upon irradiation at λ > 600 nm, an emission quenching occurs, that can only originate from a local temperature increase. The corresponding heat must, therefore, originate from the photo-thermal effect of the gold nanoshell upon light irradiation with λ > 600 nm. Huang et al. [58] studied local heating produced by laser irradiation of gold nanoparticles in the interior of a cell, both experimentally and theoretically. It was found that, for a 50 mW laser power focused into a 0.1 mm spot, the temperature at the vicinity of gold nanoparticles would increase from 25 °C to approximately 38 °C, after 4 min of irradiation and for an absorption at the plasmon resonance wavelength of 0.1. Using a light power meter (Thorlabs PM100USB with a S140C sensor), the light intensity through the λ > 600 nm filter was ~5 mW. Assuming 50% loss upon the used coupling to an optical fiber with a 1-mm diameter, and considering the final irradiation time of 120 min, the equivalent light power in the conditions of the calculations of Huang et al. [58] would be ~18 mW. According to the reported linear relation between the calculated temperature near gold nanoparticles and irradiation time [58], a local temperature increase of ~5 °C is expected. This temperature increase is compatible with the observed rhodamine fluorescence quenching. A much higher local temperature increase using laser irradiation of the developed magnetic/plasmonic lipid covered systems is then expected.

Therefore, these results show that solid magnetoliposomes based on manganese ferrite/gold core/shell nanoparticles are promising agents for plasmonic photothermal therapy. The local temperature increase caused by irradiation of the core/shell nanoparticles will also promote an increase in fluidity of the lipid membrane of liposomes, which enhances drug release.

## 4. Conclusions

In this work, liposomes containing magnetic/plasmonic nanoparticles with manganese ferrite core and a gold shell were prepared. The multifunctional liposomes obtained, with sizes around or below 150 nm, were revealed to be suitable nanocarriers for an anticancer drug (a thienopyridine derivative), exhibiting high encapsulation efficiencies. Drug-loaded aqueous magnetoliposomes (with the nanoparticles entrapped in liposomes) were able to interact with model membranes by fusion. The solid magnetoliposomes (where the core/shell nanoparticles are covered by a lipid bilayer) have shown the ability to be used in photothermia applications. Therefore, the multifunctional liposomes containing MnFe_2_O_4_/Au core/shell nanoparticles were found to be promising agents for combined chemo/phototherapy.

## Figures and Tables

**Table 1:** Selected Rietveld analysis parameters.

Sample	O_x,y,z_ (*)	*i* (*)	Micro Absorption Correction	Overall Temperature Factor, B_over_	Lattice Constant (nm)	Size (nm)	R_f_	χ^2^
**MnFe_2_O_4_**	0.251	0.928	No	0 (^+^)	0.84693	13.8	9.03	1.33
**MnFe_2_O_4_**	0.251	0.898	No	−2.79	0.84684	13.2	4.45	1.18
**MnFe_2_O_4_**	0.257	0.60	Yes (#)	0 (^+^)	0.84685	13.3	3.18	1.18
**MnFe_2_O_4_/Au**	0.257 (^+^)	0.60 (^+^)	Yes (##)	0 (^+^)	0.84685 (^+^)	13.3	4.70	1.56
	---	---		0 (^+^)	0.406945	11.7	0.68	

(#) P_0_ = 0.629, C = 1.31, τ = 0.055. (##) P_0_ = 0.607, C = 1.15, τ = 0.084. (^+^) fixed values. (*) Values in CIF file 2360018 are O**_x,y,z_** = 0.25053 and *i* = 0.33.

**Table 2:** **C**oercive field (*H*_c_), saturation magnetization (*M*_s_), remnant magnetization (*M*_r_), and ratio *M*_r_/*M*_s_ for MnFe_2_O_4_/Au core/shell nanoparticles at room temperature.

	*H*_c_ (Oe)	*M*_s_ (emu/g)	*M*_r_ (emu/g)	*M*_r_/*M*_s_
MnFe_2_O_4_/Au NPs	13.57	3.15	0.08	0.03

**Table 3:** Steady-state fluorescence anisotropy (*r*) values, at 25 °C, for the anti-tumor compound in magnetoliposomes in comparison with the values in neat liposomes.

	Lipid	*r*
**Liposomes** [50]	Egg-PC	0.176
	DOPG	0.181
**AMLs**	Egg-PC	0.173
	DOPG	0.168
**SMLs**	DOPG	0.175
